# The complete mitochondrial genome of *Bothrops jararaca* (Reptilia, Serpentes, Viperidae)

**DOI:** 10.1080/23802359.2016.1149783

**Published:** 2016-12-09

**Authors:** Diego Dantas Almeida, João Paulo Kitajima, Milton Yutaka Nishiyama, George Willian Condomitti, Ursula Castro de Oliveira, João Carlos Setúbal, Inácio L. M. Junqueira-de-Azevedo

**Affiliations:** a Instituto Butantan, Lab. Especial de Toxinologia, São Paulo, Brazil;; b Mendelics Análise Genômica, São Paulo, Brazil;; c Instituto de Química, Universidade de São Paulo, São Paulo, Brazil

**Keywords:** *Bothrops jararaca*, Mitogenome, Mitochondrial DNA

## Abstract

The complete mitochondrial genome, containing 17,526 bp, was determined from the pitviper *Bothrops jararaca*. It is the first mitogenome for the most medically important genus of snake in Latin America. This mitogenome has common snake mitochondrial features such as a duplicated control region that has nearly identical sequences at two different locations of the mitogenome and a translocation of tRNA-Leu (UUR). Besides, we found a translocation of the tRNA-Pro compared to Colubridae snakes. Finally, an unusual possible duplication containing a tRNA-Phe was observed for the first time and may represent a marker of the genus.

*Bothrops jararaca* belongs to Viperidae family and is distributed mainly in the Southeast and South Regions of Brazil. It is the most medically important snake in South America, and its venom is one of the most studied among all snakes (Cardoso 1990, Goncalves-Machado et al. 2015). Distinct populations are characterized by a high genetic diversity, whereas sister species such as *B. insularis* and *B. alcatraz* have some genetic proximity to certain populations (Grazziotin et al. 2006). However, no complete mitochondrial genome is available for the genus, which encompasses up to 44 species, most of them of high medical interest.

Here, we determined the complete mitogenome of *B. jararaca* (GenBank accession number KU194299) by whole genome shotgun using Illumina paired-end sequencing (Illumina HiSeq1500). The specimen was collected at Embu das Artes, São Paulo State, Brazil – Geospatial coordinates: −23.654919, −46.864827 – and is disposed at the Herpetological Collection of the Special Laboratory of Zoological Collections from Butantan Institute (accession number: IBSP 84406). The genes were predicted by two independent softwares (MITOS and DOGMA) and were manually curated. A phylogenetic tree with the closest species available is shown in Figure 1. The gene order and structure is similar to that found in the family (Dong & Kumazawa 2005; Huang et al. 2014). The total length of this mitogenome is 17,526 bp and it contains 13 protein-coding genes (PCGs), 2 rRNA genes, 23 tRNA genes (including a possible new tRNA which has 88% of sequence similarity to tRNA-Phe), and 2 control regions. Most genes are coded on the heavy strand (H-strand) except for the *ND6* and 8 *tRNAs*. The base composition of the light strand is 31.9% A, 24.5% T, 13.3% G, and 30.3% C. The high AT content agrees with that of other snakes (Jang & Hwang 2011; Li et al. 2014).

**Figure 1.**
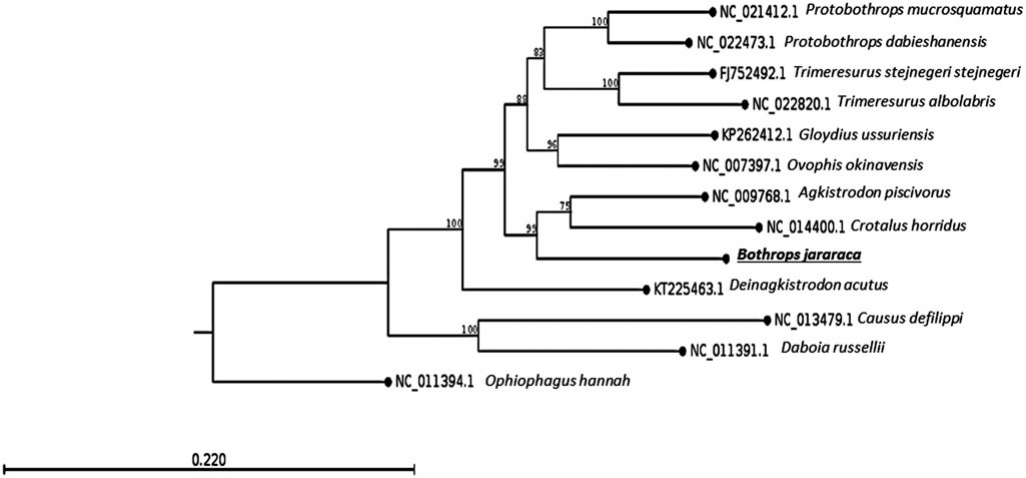
The maximum-likelihood phylogenetic tree of *B. jararaca* mitocondrial DNA and 11 closely related species (Viperidae) whose mitogenome sequences have been reported, indicates the placement of *B. jararaca* genome in the Crotalinae subfamily, closely to the sister taxa (Crotalus and Agkistrodon) of genus Bothrops. The tree was generated using 1000 replicates and the boostrap values are shown on each node. The mitogenome of *Ophiophagus hannah* (Elapidae) was used as an outgroup.

The mitogenome of *B. jararaca* contains almost identical duplicate regions corresponding to the Control Regions (CRs). CRI is located between *tRNA-Thr* and *tRNA-Ph*e genes and CRII is located between the *tRNA-Pro* and *tRNA-Leu* genes. As in other snakes, the *tRNA-Leu* (UUR) gene, which occurs between the *16S rRNA* and *ND1* genes in other vertebrates, is located downstream of the CRII. As in other *Viperidae* snakes (Jiang et al. 2007; Hall et al. 2013; Huang et al. 2014), the *tRNA-Pro* gene is located between *CRII* and *tRNA-Ile* gene instead of the *CRI 5′* vicinity (a pseudo *tRNA-Pro* gene can be found between *CRII* and *tRNA-Ile* gene in *Colubridae*) (Jang & Hwang 2011). The predicted origin of L-strand replication (OL) is 35 bp in length and is located between the tRNA-Asn and tRNA-Cys.

A distinctive feature in the *B. jararaca* mitogenome is the presence of a possible tRNA-Phe duplication, with 88% identity. The *tRNA-Phe*-like gene is found downstream of the CRI region. We consider the *tRNA-Phe* coded in the position 1 to 65 as the functional one; the role of the other one needs to be determined. It will remain to be investigated if this distinctive feature is conserved among other related species, but if it is, the synapomorphy may be a useful character to help in elucidating the controversial phylogeny of the group.

This work presents the first mitogenome of a *Bothrops* species, showing that it has both similar and distinctive features when compared to those from other genera, providing additional molecular data for phylogenetic studies of snakes.
